# Bone Marrow Adipose Tissue

**DOI:** 10.3390/cells13090724

**Published:** 2024-04-23

**Authors:** Elena Marinelli Busilacchi, Erika Morsia, Antonella Poloni

**Affiliations:** 1 Hematology Laboratory, Department of Clinical and Molecular Sciences, DISCLIMO, Università Politecnica delle Marche, 60126 Ancona, Italy; e.busilacchi@staff.univpm.it (E.M.B.); e.morsia@univpm.it (E.M.); 2 Hematology, AOU delle Marche, 60126 Ancona, Italy

**Keywords:** bone marrow microenvironment, bone marrow adipocytes, bone marrow disorder

## Abstract

Bone marrow (BM) acts as a dynamic organ within the bone cavity, responsible for hematopoiesis, skeletal remodeling, and immune system control. Bone marrow adipose tissue (BMAT) was long simply considered a filler of space, but now it is known that it instead constitutes an essential element of the BM microenvironment that participates in homeostasis, influences bone health and bone remodeling, alters hematopoietic stem cell functions, contributes to the commitment of mesenchymal stem cells, provides effects to immune homeostasis and defense against infections, and participates in energy metabolism and inflammation. BMAT has emerged as a significant contributor to the development and progression of various diseases, shedding light on its complex relationship with health. Notably, BMAT has been implicated in metabolic disorders, hematological malignancies, and skeletal conditions. BMAT has been shown to support the proliferation of tumor cells in acute myeloid leukemia and niche adipocytes have been found to protect cancer cells against chemotherapy, contributing to treatment resistance. Moreover, BMAT’s impact on bone density and remodeling can lead to conditions like osteoporosis, where high levels of BMAT are inversely correlated with bone mineral density, increasing the risk of fractures. BMAT has also been associated with diabetes, obesity, and anorexia nervosa, with varying effects on individuals depending on their weight and health status. Understanding the interaction between adipocytes and different diseases may lead to new therapeutic strategies.

## 1. Introduction

Bone marrow (BM) is a unique organ contained in bone cavities responsible for hematopoiesis, the regulation of skeletal remodeling, and immune regulation. The composition of BM is dynamic, as the mixture of cellular components and connective tissue shifts with age and in response to systemic factors. In humans, bone marrow is commonly referred to as “red” or “yellow” marrow, depending on the prevalence of hematopoietic or adipose cells: yellow bone marrow consists predominantly of adipocytes, comprising about 95% of the marrow, whereas red marrow consists of 20–40% adipocytes surrounded by hematopoietic cells [1].

**Figure 1:**
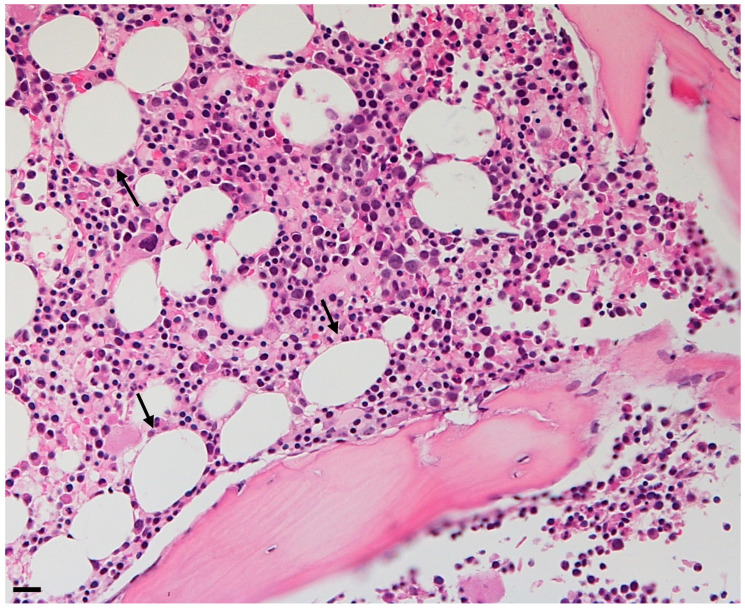
Bone marrow adipose tissue trephine of a 50-year-old woman. Adipocytes are shown as holes in the biopsy. Original magnification ×10, scale bar 20 μm.

**Figure 2:**
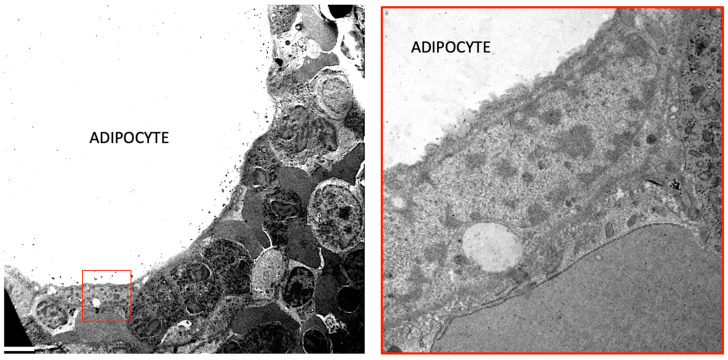
A Transmission Electron Microscopy of human normal bone marrow. **Left**: an adipocyte is surrounded by different types of hematopoietic cells. The squared area is enlarged in the **right** panel showing the nucleus of the adipocyte and the close connection with hematopoietic cells. Bar: 3 mm in left panel and 0.5 mm in right panel.

Bone marrow adipose tissue (BMAT) is localized in the bone marrow niche and presents characteristics mostly with white adipose tissue (WAT) and brown adipose tissue (BAT) in terms of mixed morphology and overlapping, but different marks comparing both [2]. These adipocytes are not clustered in lobules as in other fat depots; instead, they are dispersed within the hematopoietic tissue (Figure 1). The mean diameter of these adipocytes is typically smaller than the diameter of subcutaneous or visceral adipocytes, generally ranging from 40 to 65 µm. However, significant variations are reported in the literature depending on the location (Figure 2) [3].

The distribution of BMAT throughout the human body is heterogeneous. In adults, BMAT is readily observed in the long bones of the upper and lower limbs, as well as in the sternum, ribs, vertebral bones, sacrum, and pelvis [4,5]. In mice, BMAT is located in the red marrow of the tibia proximal to the fibula junction, in the femur, and in the axial skeleton [6].

BMAT is found inside the bone cavity together with trabecular bone, nerve fibers, blood vessels, sinusoidal capillaries and many different types of cells, including lymphoid, myeloid, and erythroid cells, that constitute the hematopoietic lineage or red bone marrow, as well as mesenchymal, otherwise called yellow bone marrow. 

At birth, most of the bone marrow is constituted by actively hematopoietic red marrow; newborns show an almost full totality of red bone marrow [7].

Over time, there is a significant decrease in red marrow, with BMAT accounting for more than 70% of the marrow space by the age of 25 years old. Subsequently, the red marrow undergoes transformation into yellow marrow at a slower rate, persisting throughout life [8]. In general, expansion of BMAT starts in the epiphyses of long bones, and proceeds in the diaphysis, progressing distally and proximally. The red marrow shrinks and gradually vacates most of the space, until being present only in the ribs, sternum, some vertebrae, sacrum, and coccyx around the age of 40 years old [9,10]. 

Gender plays a significant role in the accumulation of yellow bone marrow with aging. In female vertebrates, the absolute amount of BMAT surpasses that of age-matched males by approximately 10%, primarily due to a higher adipose content in BMAT. Furthermore, the onset of menopause leads to a reorganization in the ratio between yellow and red marrow in females due to estrogen deficiency and decreased hematopoietic activity [11,12,13,14].

## 2. Bone Marrow Adipose Tissue Embryological Origin

Less is known about the embryonic origin of BMAT. The lineage of marrow adipocytes does not follow the known developmental pathways observed for other bone marrow cell types, at least not until the final stages of differentiation [15]. Adipose tissue is commonly considered to have both mesodermal and neuroectodermal origins [16]. However, precise lineage tracing studies have not been extensively conducted, except for BAT [17].

In the BM, BMAT develops from mesenchymal stem cells (MSCs) by a progressive pathway of differentiation [15]; however, mature bone marrow adipocytes, when subjected to physiological stimuli, typically retain their lineage properties and exhibit limited transdifferentiation into completely different phenotypes, unlike what is observed in white and brown adipocytes [18,19,20,21].

The microenvironment, rather than cellular commitment alone, can be crucial in maintaining or adapting a specific cellular phenotype to physiological challenges. In vitro, mature bone marrow adipocytes slowly delipidate, expelling their lipid droplets and transforming into fibroblast-like cells with bone marrow mesenchymal stem cell properties [18,22]. In vitro studies report that BMAs express stem cell markers like Sox17, Gata4, and Oct4, but Nanog or Sox2; this could indicate that these cells have fewer plastic properties than other types of adipocytes [18].

Within the category of small non-coding RNAs, microRNAs (miRNAs) provide an additional mechanism for regulating the expression of genes involved in adipogenesis [23]. Due to their remarkable ability to simultaneously govern multiple protein targets and biological processes, it has been proposed that miRNAs could play a pivotal role in the differentiation of bone marrow-derived stem cells. Researchers have explored their involvement in adipogenesis through both experimental and target-focused computational approaches [24,25]. Some identified miRNAs seem to influence the commitment of bone marrow stromal cells (BMSCs) to become adipocytes, while others affect their maturation into mature fat cells. Notably, in some cases, the latter group of miRNAs seems to act as switches, inhibiting the osteogenic process and promoting the adipogenic process [26,27].

Giuliani et al. have shown that miR-422a and miR-483-5p act as pro-differentiation factors in BMSCs and that these two miRNAs can affect the adipogenesis process by influencing Methyl-CpG binding Protein 2 (MeCP2) expression. These findings underscore the importance of elucidating MeCP2 expression and regulation in peripheral tissues, particularly in bone marrow stromal cells, to understand their potential relevance to associated diseases [28].

## 3. Types of Bone Marrow Adipose Tissue

Two types of BMAT can be distinguished: constitutive (cBMAT) and regulated (rBMAT) adipose tissue. cBMAT is characterized by being more stable and less reactive yellow tissue. In contrast, rBMAT is immersed in red marrow and continuously undergoes modification to respond to nutritional, hormonal, and temperature changes.

Moreover, both types of marrow adipocytes exhibit different positional characteristics. cBMAT develops early in life in mice and is located at the distal tibia and caudal vertebrae of the tail. In contrast, rBMAT develops during life and is strain-specific, distributed within active hematopoietic niches such as the mid- to proximal tibia, femur, and lumbar vertebrae.

Furthermore, compared to cBMAT, which consists of larger, round adipocytes that primarily store unsaturated fatty acids (mostly palmitoleic or myristoleic acid) and exhibit higher resistance to cold exposure, rBMAT adipocytes are smaller and contain more saturated fatty acids that can be readily mobilized upon stimulation, similar to WAT [29].

rBMAT is more prone to regulation: in certain scenarios, such as estrogen deficiency, high-fat diet-induced obesity, or caloric restriction, rBMAT can expand extensively, even if both tissues are increased [14,30,31].

A latest theory proposes that the rBMAT is formed at first and finally matures into cBMAT, but still, the relationship between these two tissues is not clarified [32].

When comparing the gene expression profiles of rBMAT and cBMAT, it has been found that the expression level of peroxisome proliferator-activated receptor gamma (PPARγ) is similar between the two types. However, the levels of CCAAT-enhancer-binding proteins α and β (Cebpα and Cebpβ) are lower in rBMAT adipocytes [6,33].

Transcriptome analysis of BMAT has revealed a metabolic profile associated with leukocyte differentiation and bone remodeling. BMAT exhibits lower expression levels of insulin receptor precursor, insulin receptor substrate-1, and glucose transporter type 4 compared to WAT. This translates to insulin resistance and reduced glucose uptake under cold conditions [34].

BMAT has a high capability of storing lipids, similar to WAT: marrow adipocytes express the insulin receptor and are insulin-sensitizing, storing fatty acid that can be metabolized to produce ATP through the tricarboxylic acid cycle or via Wnt signaling for osteoblasts [35,36].

Moreover, cBMAT is more susceptible to β3-receptor agonist-stimulated lipolysis or cold compared with rBMAT [37]. Furthermore, it is worth noting that the composition of fatty acids in cBMAT contains a higher concentration of unsaturated fatty acids, such as palmitoleate and oleate, compared to those found in rBMAT [6].

## 4. Bone Marrow Adipose Tissue Functions in Health

Bone marrow adipose tissue is regarded as a central component that plays various roles in bone marrow homeostasis. It influences bone health, density, and remodeling, as well as alters hematopoietic stem cell (HSC) functions. Additionally, it contributes to the commitment of mesenchymal stem cells (MSCs) and influences immune cell function within the bone marrow microenvironment, thereby contributing to immune homeostasis and defense against infections. Moreover, it participates in energy metabolism and inflammation. For instance, when BMAT is more saturated, it is associated with reduced trabecular bone mineral density and an increased risk of experiencing new vertebral fractures. Conversely, when BMAT is less saturated, it is associated with more favorable outcomes [38].

Additionally, bone marrow adipose tissue releases specific signaling molecules that influence the bone marrow environment and the process of bone remodeling. One example of such a molecule is the receptor activator of NF-κB ligand (RANKL), which is found in higher concentrations in BMAT compared to white adipose tissue (WAT) and brown adipose tissue (BAT). When RANKL is removed from BMAT, it inhibits bone resorption while leaving bone formation unaffected. This safeguard mechanism protects mice against the loss of trabecular bone caused by ovariectomy and rosiglitazone [39].

In bone marrow, the differentiation of bone marrow adipose tissue (BMAT) from MSCs is more directly correlated to osteoblasts than to other cells such as marrow stromal cells, chondrocytes, and myocytes. This shared origin leads to a competition between alternative differentiations towards osteogenesis or adipogenesis. The adipogenesis within the medullary environment promotes the formation of adipocytes while actively suppressing osteogenesis [40,41]. Reciprocal regulation of mesenchymal cell fate may explain the reduction of bone mass and expansion of BMAT with age [10], but the exact signaling pathways involved have not been elucidated.

Bone marrow adipose tissue secretes adipokines and fatty acids that can stimulate the activation and differentiation of osteoclasts. Several in vitro studies utilizing mature osteoblasts from trabecular bone or bone marrow stromal cells have demonstrated the reversible conversion of these cells between adipogenic and osteogenic lineages in response to external factors [42,43].

In vitro experiments demonstrate that bone marrow adipocytes support myeloerythroid differentiation, and the administration of PPARγ agonists increases marrow adipogenesis and reduces leukemia progenitor cells [44,45,46]. PPARγ is also involved in the regulation of energy balance and lipid homeostasis. It is necessary to initiate adipogenesis and inhibit any other type of differentiation pathway [40,47].

Adipose-specific PPARγ knock-out mice displayed the absence of fat tissue even under conditions of a high-fat diet, confirming the significance of these factors in triggering adipogenesis and maintaining the differentiation of bone marrow stem cells [48].

PPARγ also improves the production of several adipokines secreted by adipocytes, including leptin and adiponectin.

Adiponectin is an adipokine involved in various metabolic processes, including whole-body energy homeostasis and glucose metabolism. It exerts diverse systemic effects on vascular function, insulin sensitivity, and anti-inflammatory responses [49,50]. This hormone might be a negative regulator of bone metabolism, because it is inversely correlated with bone mineral density. Adiponectin also negatively influences adipogenesis, as has been shown in a long-term human bone marrow culture or in transgenic mice [29,51,52]. Adiponectin also exhibits osteogenic actions by promoting the osteogenic differentiation of human bone marrow MSCs and human adipose-derived stem cells [53,54].

Leptin is one of the principal protein hormones produced by adipocytes and regulates energy homeostasis, food intake, and bone formation and resorption via osteoblast proliferation [55,56]. Leptin also regulates the differentiation of bone marrow mesenchymal stromal cells into osteoblasts or adipocytes via the leptin receptor (LepR). In mice, deficiency or absence of LepR leads to decreased bone mass and bone length, along with a parallel increase in bone marrow adiposity [56,57,58]. In addition, serum leptin concentration is directly correlated with body fat stores [59].

The role of BMAT in the production of hematopoietic cells is a topic of debate. On the one hand, expansion of BMAT is generally associated with decreased hematopoiesis. On the other hand, stem cell factor (SCF) produced by the adipocytes supports HSC survival and self-renewal [45,60]. Moreover, adiponectin supports HSCs, but reduces B cell differentiation promoting myelopoiesis [61]. BMAT forms a fundamental part of the hematopoietic stem cell niche. It provides mechanical and metabolic support to HSCs, thereby contributing to their proliferation, self-renewal, and maintenance [62].

BMAT secretes adipokines and growth factors, including adiponectin and leptin, which influence the fate and function of hematopoietic cells. Marrow adipocytes have been demonstrated to promote hematopoietic stem cell survival and support the regeneration of HSCs following irradiation or chemotherapy treatment through the secretion of stem cell factors [46,63].

Mattiucci et al. demonstrate that bone marrow adipocytes can support and maintain the survival and development of HSCs in an in vitro long-term culture-initiating cell system, and at the same time BMAs produce cytokines implicated in hematopoiesis regulation [44].

BMAT can also modulate the supply of energy-rich molecules to hematopoietic cells, depending on the metabolic needs of the body, thus influencing hematopoiesis [60,62,64].

Adipokines released by BMAT contribute to the development of inflammaging, characterized by chronic low-grade systemic inflammation. This process disrupts the balance between anti-inflammatory and pro-inflammatory stimuli, affecting network functions such as metabolism and energy usage. Additionally, various cytokines produced by BMAT, such as TNF-α, IL-6, and CCL2/MCP-1, play crucial roles in inflammation-driven bone loss. The age-related increase in adiposity within the bone marrow exacerbates inflammaging and bone loss, leading to alterations in bone structure and impairments in HSC health and functions. Furthermore, the shift from bone marrow stromal cells to bone marrow adipocytes increases reactive oxygen species (ROS) production, weakening the potential of HSCs, promoting cellular senescence, and disrupting normal immune system responses, thereby enhancing myelopoiesis [65].

## 5. Bone Marrow Adipose Tissue Functions in Diseases

BMAT has been identified as a central contributor to the pathogenesis and progression of various diseases. This understanding has sparked new avenues of research, elucidating the intricate relationship between BMAT and health, particularly in hematological malignancies, skeletal conditions, and metabolic disorders.

Studies have shown that BMAT promotes the growth of tumor cells in acute myeloid leukemia (AML), with a reduction in marrow adipocytes correlating with an increase in tumor cells. Notably, both laboratory experiments and research involving living organisms demonstrate that leukemia cells prompt functional changes in non-cancerous adipocytes. This leads to the transfer of free fatty acids (FAs) from adipocytes to AML blasts, with fatty acid binding protein-4 (FABP4) playing a role in this process. Suppressing FABP4 in adipocytes significantly impedes AML survival in co-culture setups, underscoring the significance of adipocyte-derived FABP4 in AML maintenance. Moreover, reducing FABP4 in AML cells enhances survival rates in animal models [66]. Additionally, the administration of PPARγ agonists in a xenograft model represses leukemic growth, induces bone marrow adipogenesis, and rescues healthy hematopoietic maturation [47].

Furthermore, meta-analysis studies have demonstrated that obesity, which is linked to heightened bone marrow adiposity, is associated with an increased susceptibility to leukemia development and elevated mortality rates. The precise biological mechanism connecting overweight and obesity to the heightened leukemia risk remains unclear. One metabolic consequence of obesity is insulin resistance, which results in increased insulin secretion from the pancreas. Insulin may potentially directly stimulate tumor development by interacting with insulin receptors on (pre)neoplastic cells or indirectly by elevating levels of insulin-like growth factor-I (IGF-I). Both normal and cancerous hematopoietic cells express receptors for IGF-I, which not only contributes to hematopoiesis but also acts as a growth factor for myeloid and lymphoid leukemia cell lines. Furthermore, the elevated leukemia risk in obese individuals could also be attributed to compromised immune function and chronic inflammation associated with obesity. Lastly, adipocytes in adipose tissue contribute to inflammation by activating T cells and macrophages, thus intensifying local inflammation in the bone marrow microenvironment [67,68].

The dysregulation of adipocytes within the bone marrow microenvironment has been linked to a range of bone marrow disorders, including multiple myeloma (MM). Bone marrow adipocytes excrete numerous free fatty acids, adipokines, and growth factors (such as IGF-1 and VEGF) that promote myelomagenesis within the bone marrow, facilitating further disease progression [69]. Niche adipocytes protect cancer cells against chemotherapy, trigger autophagy through increasing the expression of autophagic proteins, and decrease apoptosis in myeloma cells [70,71]. A study conducted in the USA using in vitro methods demonstrates that adipocytes in the bone marrow trigger autophagy in MM cells through the action of adipsin and leptin adipokines. This activation of autophagy subsequently leads to resistance to chemotherapy. Several signaling pathways are involved in regulating autophagy, including Akt/mTOR and Bcl-2, which inhibit the accumulation of autophagy proteins, while JNK and NF-κB promote the transcription of genes related to autophagy. Additionally, Jak/Stat3 signaling, which is activated by adipokines secreted by adipocytes, enhances the expression of specific autophagy-related proteins. However, the exact mechanism underlying this process requires further investigation [72].

Moreover, MM cells can change the methylation pattern of PPARγ in BMAs, contributing to bone disease, increasing osteoclast formation, and inhibiting osteoblast generation [73].

In the bone microenvironment, the interaction between adipocytes and tumor cells is a dynamic and complex process that can influence tumor progression and therapy outcomes. Understanding the interaction between adipocytes and leukemia cells in the bone marrow may lead to innovative therapeutic strategies for the treatment of hematological malignancies. For example, disrupting the protective niche created by adipocytes might be useful to sensitize leukemia cells to chemotherapy [62,74,75].

Several pieces of evidence have shown that BMAT may play a critical role in the development or progression of other human tumors and in skeletal metastases. In prostate cancer, BMAT promotes bone marrow metastasis by increased the expression of fatty acid binding protein-4. Herron et al. have demonstrated that up-regulation of FABP4 helps cancer cells acquire fatty acids from BMAT, permitting them to grow within the bone microenvironment [76]. Similar observations have been made in ovarian cancer, where FABP4 is more prevalent in low-grade tumors compared to high-grade ones. In high-grade carcinomas, FABP4 is primarily expressed in stroma-rich regions, potentially fostering the development of a more mature vasculature and resistance to anti-VEGFA therapy. Silencing FABP4 in blood vessels, while preserving its expression in adipose tissue, results in decreased vessel density and tumor growth, underscoring its significant role in tumor angiogenesis [77].

In postmenopausal women with breast cancer, there is a positive correlation between bone marrow adipose tissue (BMAT), tumor size, and histological grading. Magnetic resonance spectroscopy studies have shown in vivo imaging displaying a positive association between the amount of bone marrow fat and breast cancer incidence and severity. However, blood leptin levels do not display this correlation [78].

The role of adipocytes in the bone marrow microenvironment can indeed impact bone density and remodeling, potentially leading to conditions like osteoporosis [79,80]. High levels of bone marrow adiposity are inversely correlated with bone mineral density, and this association can contribute to the development of osteoporosis. Osteoporosis is characterized by fragile bones and an increased risk of fractures [6,31,81].

RANKL plays a crucial role in regulating osteoclast-mediated bone remodeling, as it stimulates osteoclast formation. Disruptions in RANKL levels are associated with bone loss and an increased susceptibility to fractures. In rheumatoid arthritis (RA), RANKL expression is heightened in the synovial tissue. Blocking RANKL using denosumab, a neutralizing antibody against RANKL, has shown promise in mitigating bone damage in RA, particularly in the short term. Functional deficiency of RANKL significantly slows down the progression of arthritis, evidenced by diminished clinical arthritis symptoms and synovial tissue overgrowth. Surprisingly, mice lacking functional RANKL exhibited higher survival rates compared to control mice, hinting at a potential compensatory role for other members of the tumor necrosis factor superfamily in the absence of RANKL [82]. 

In postmenopausal osteoporotic women with estrogen deficiency, there is often a notable increase in marrow adiposity alongside heightened production of sclerostin (SOST) from osteogenic cells. Interestingly, there exists a positive correlation between cortical bone matrix levels of SOST and Dickkopf-1 (DKK1) and bone mass and strength. Experimental evidence suggests that DKK1 suppresses osteoblast function and mineralization, contributing to osteopenia, while SOST inhibits osteoblast differentiation and function [83]. Additionally, estrogen deficiency stimulates in vitro transdifferentiation of pre-osteoblast cell lines into adipocytes via down-regulation of β-catenin [84]. Finally, estrogen therapy in postmenopausal women results in a decrease in BMAT, indicating that this hormone exerts regulatory action on bone marrow cells [13]. 

Changes in BMAT have been observed beyond that of primary osteoporosis and in various other systemic conditions, including obesity, anorexia nervosa, and diabetes [85]. 

Obesity is a multifactorial chronic disease characterized by metabolic abnormalities, immune dysfunction, oxidative stress, and chronic low-grade inflammation [86,87]. While it has been demonstrated that WAT responds to overnutrition by mounting an immune response, the contribution of BMAT to the metabolic and inflammatory complications of obesity remains somewhat debated. Studies employing high-fat feeding mouse models have demonstrated an augmentation in BMAT formation and adipocyte differentiation of mesenchymal stem cells derived from the bone marrow. Concurrently, this increase in BMAT is accompanied by a reduction in bone mass. Surprisingly, BMAT retained its responsiveness to insulin and did not display a pro-inflammatory phenotype or insulin resistance, which contrasts with the behavior observed in peripheral adipose tissue [88].

Women with anorexia nervosa have low subcutaneous and visceral WAT (vWAT) and more BMAT compared with normal-weight controls, as measured by magnetic resonance imaging. The association between higher bone marrow fat content and lower bone mineral density in these women suggests weakened bones. However, the underlying reasons for this abnormal increase in bone marrow fat are not yet fully understood [81,89,90,91].

In overweight/obese women, vWAT and BMAT are positively correlated [92]. In overweight and obese men, there is an inverse relationship between bone mineral density and BMAT: individuals with larger visceral adipose tissue (VAT) deposits demonstrate compromised bone microarchitecture and mechanical properties compared to overweight and obese men with smaller VAT deposits. This suggests that excessive accumulation of VAT negatively impacts bone health [91,93].

An increased bone marrow adipose tissue (BMAT) has been reported in both type 1 diabetes (T1DM) and type 2 diabetes (T2DM). Mouse models of both T1DM and T2DM have consistently demonstrated lower bone mass and higher marrow adiposity compared with controls. In vivo, insulin deficiency and hyperglycemia are linked to reduced expression of RUNX2 and its downstream targets, which are essential for osteoblast development. Significantly, insulin treatment reinstates the expression of RUNX2 and its regulated genes in regenerating bone, suggesting its potential in alleviating the detrimental effects of untreated diabetes on bone regeneration [94,95,96,97].

In contrast, human studies of T1DM show a non-significant increase in BMAT compared to the control group [98]. Young women with T1DM have been shown to have slightly higher levels of vertebral marrow fat than nondiabetic controls, and higher marrow fat levels correlate with greater visceral fat [99]. Additionally, human studies of T2DM demonstrate a non-statistically significant higher level of bone marrow fat in diabetic individuals compared to control groups [100].

Generally, in diabetes, increased triglycerides and free fatty acids activate PPARγ. Slade et al. have reported a positive correlation with vertebral and tibial BMAT and serum lipid levels in humans with type 1 diabetes [99]; however, in diabetic mice treatment with a PPARγ antagonist, BMAT was found to decrease but bone mass did not increase [101].

Aplastic Anemia (AA) is an autoimmune disease characterized by pancytopenia, severe anemia, and “fatty marrow”, indicating that bone cavities are filled with BMAT [102]. High leptin concentration has been found in the plasma and bone marrow biopsy tissue of AA patients. Additionally, in an AA mouse model, it has been shown that leptin levels increased both in the plasma and in bone marrow. Moreover, the increase in leptin is positively correlated with T cell function disorder and inversely correlated with bone marrow hematopoietic capacity [103]. It is well known that leptin is implicated in inflammation of the bone marrow microenvironment, in immune regulation, and in hematopoiesis.

## 6. Promising Frontiers and Conclusions

Since bone marrow adipocytes play a role in bone remodeling and bone density, therapies targeting the regulation of adipocytes may offer new avenues for osteoporosis treatments. For example, since the LepR displayed on MSCs in the bone marrow inhibits osteogenesis and promotes adipogenesis, it can be hypothesized that the use of LepR antagonists could be beneficial for osteoporosis therapies [104].

In a study using knock-in mice, researchers were able to track the fate of adipocytes in the regenerating niche of the bone marrow in vivo. By employing a new specific marker for BMAs, perilipin, the researchers demonstrated that mature bone marrow adipocytes can dedifferentiate into mesenchymal stromal cells and can transform into progenitors of osteolineage cells. Furthermore, after myeloablation, bone marrow adipocytes seem to play a role in the recovery of blood cells [105].

Additionally, the use of bisphenol-A-diglycidyl, a PPARγ antagonist, in diabetic mice reduces serum free fatty acid and triglyceride levels, as well as bone adiposity typical of this pathology, without impairing osteoblast gene expression and bone mineral density. This suggests that such treatment could potentially rescue bone health in diabetic individuals [101]. 

In conclusion, BMAT is a dynamic component of the bone marrow niche with diverse and complex roles in both health and disease. This review highlights the key roles of bone marrow adipocytes in physiological processes such as supporting HSCs, bone remodeling, energy metabolism, and modulation of immune function. 

Moreover, bone marrow adipocytes play a pivotal role in the development and progression of various diseases, including metabolic disorders, skeletal conditions, and hematological malignancies. The role of adipocytes in pathophysiology is an evolving field of research. It would be important to investigate the specific signaling pathways that regulate BMAT function in order to develop targeted therapies to modulate bone marrow adipocytes for the treatment and prevention of diseases.

Finally, the ability of bone marrow adipocytes to dedifferentiate and potentially transform into other cell types holds exciting implications for regenerative medicine. Besides, given the peculiar location of BMAT, it could be hypothesized to “drive” the fate of adipocytes in order to modulate the disease’s response to drugs.

Further research is needed to explore the mechanisms controlling these transformations and their potential for therapeutic applications. This area of study has the potential to revolutionize our approach to tissue regeneration and repair.
